# Supplementary figures and images for: Uncovering the ceRNA network and DNA methylation associated with gene expression in nasopharyngeal carcinoma
Source: BMC Med Genomics. 2023 Sep 14;16:218. doi: 10.1186/s12920-023-01653-1 (PMC10500855; doi:10.1186/s12920-023-01653-1)

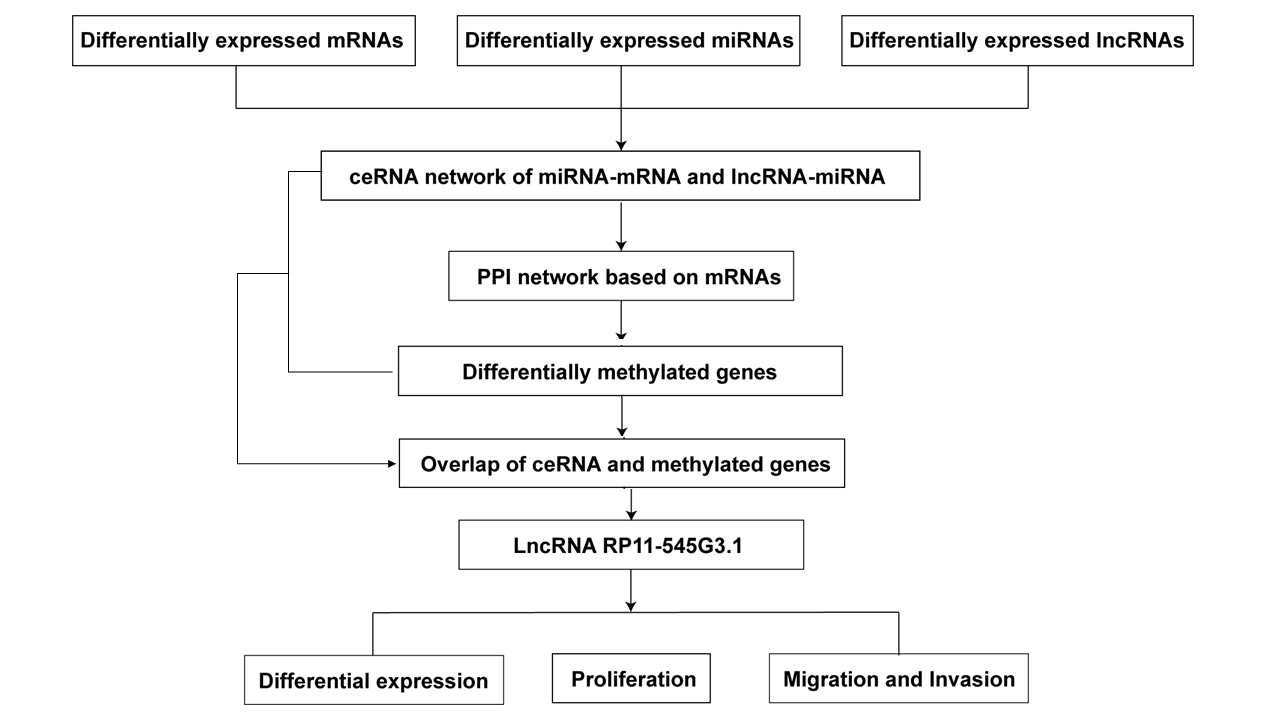

Supplement: Supplementary file 1 — Supplementary Material 1. S Fig. 1. The flowchart of differentially expressed mRNAs, miRNAs, lncRNAs and subsequent analysis [file 12920_2023_1653_MOESM1_ESM.tif]

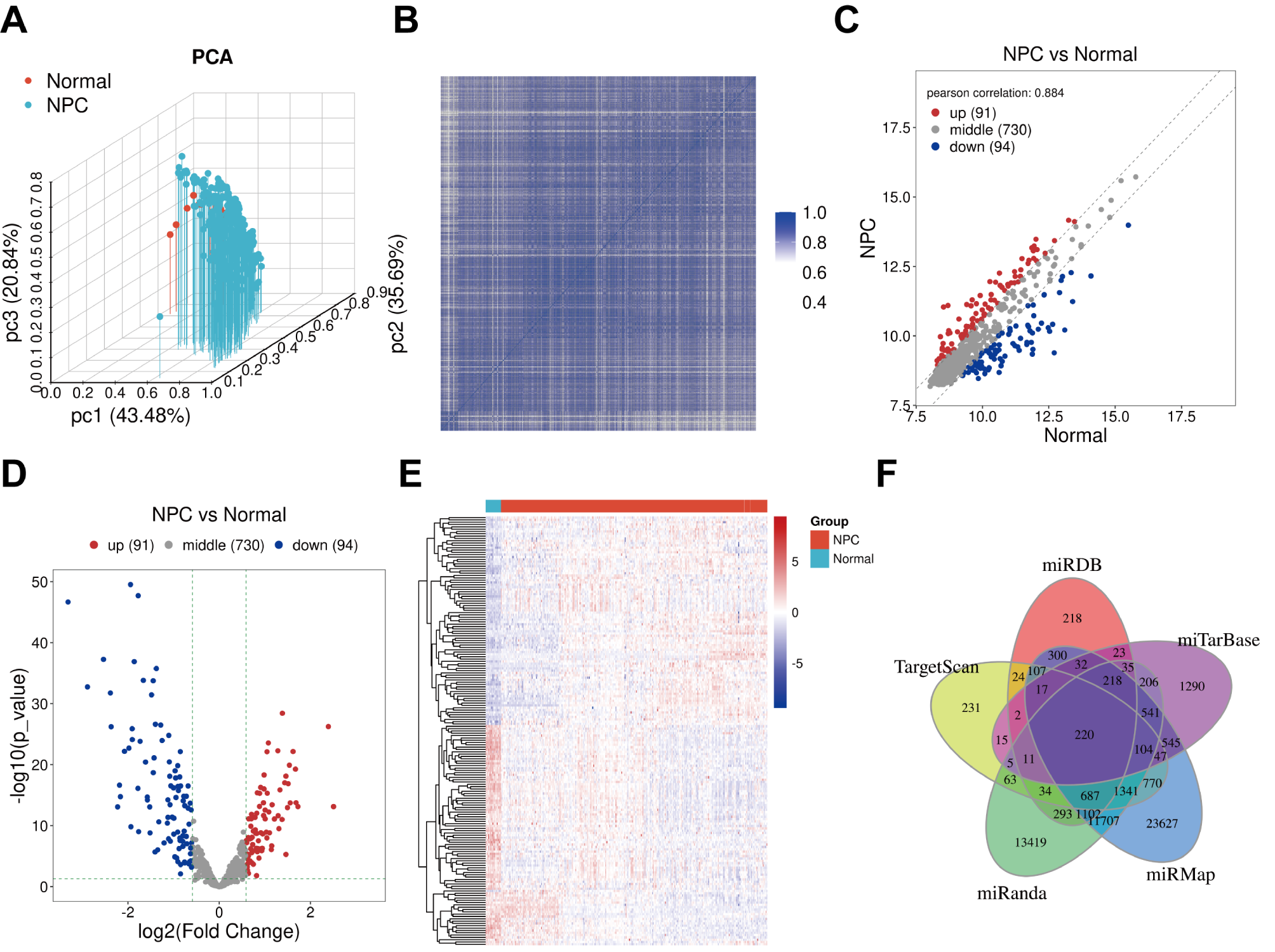

Supplement: Supplementary file 2 — Supplementary Material 2. S Fig. 2. Analysis of abnormally expressed miRNAs for nasopharyngeal carcinoma in the GSE32960 dataset. (A) PCA results of 312 nasopharyngeal carcinoma (blue) and 18 normal samples (green). (B) Heat map for the correlations between samples. (C−E) Scatter plots, volcano diagram, and heat map of the differentially expressed miRNAs between nasopharyngeal carcinoma and normal specimens. Blue: down-regulation; red: up-regulation. (F) Target mRNAs of differentially expressed miRNAs by miRTarBase, TargetScan, miRDB, miRanda and miRMap databases [file 12920_2023_1653_MOESM2_ESM.tif]

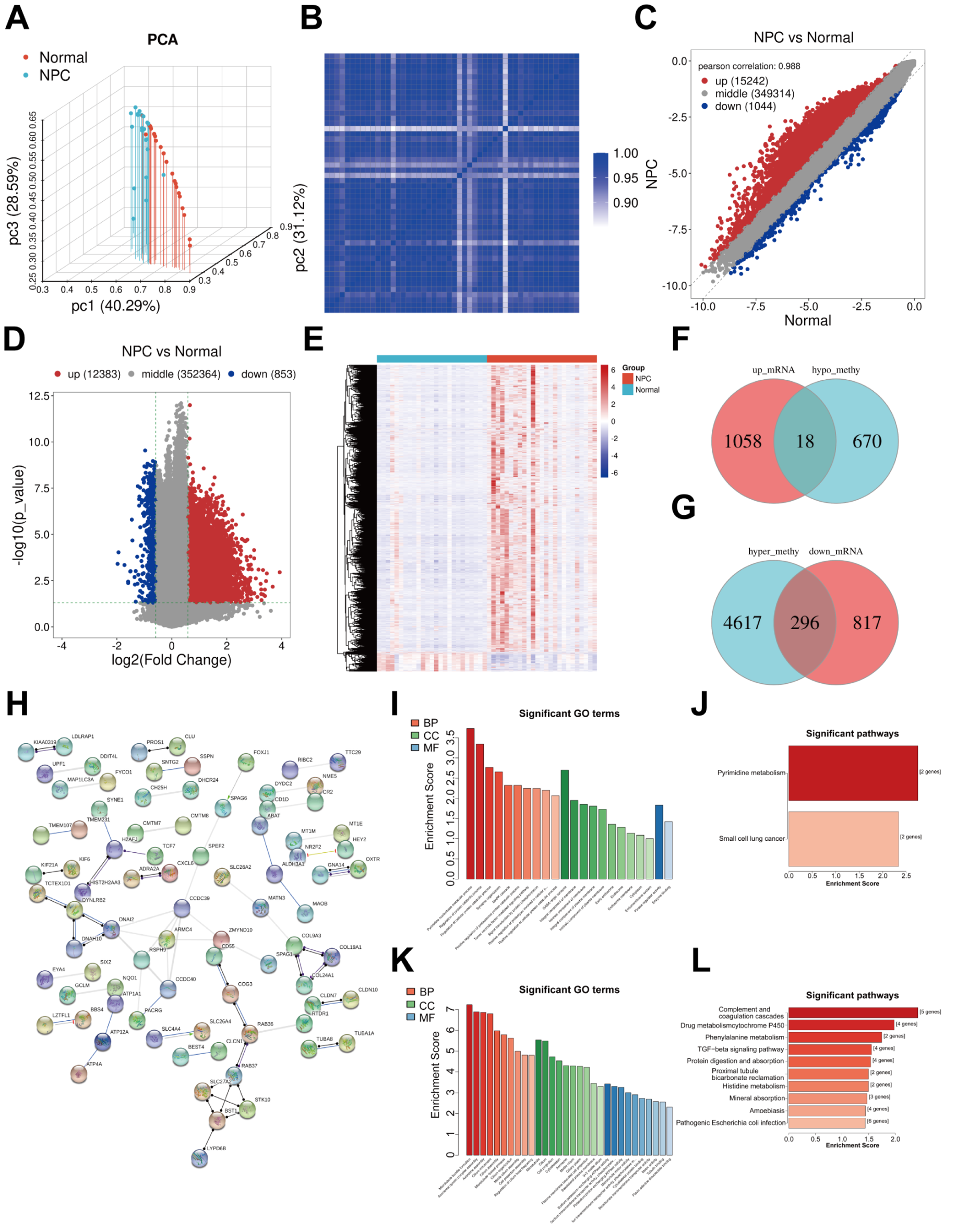

Supplement: Supplementary file 3 — Supplementary Material 3. S Fig. 3. Analysis of differentially methylated genes in nasopharyngeal carcinoma in the GSE62336 dataset. (A) PCA results of nasopharyngeal carcinoma (green) and normal samples (blue). (B) Heat map for the correlations between samples. (C−E) Scatter plots, volcano diagram, and heat map of the differentially methylated sites in nasopharyngeal carcinoma. Red: hypermethylation and blue: hypomethylation. (F) Overlap of the highly expressed genes and hypomethylated genes. (G) Overlap of the lowly expressed genes and hypermethylated genes. (H) A PPI network of lowly expressed and hypermethylated genes. (I, J) GO and KEGG enrichment results of highly expressed and hypomethylated genes. (K, L) GO and KEGG enrichment results of lowly expressed and hypermethylated genes [file 12920_2023_1653_MOESM3_ESM.tif]

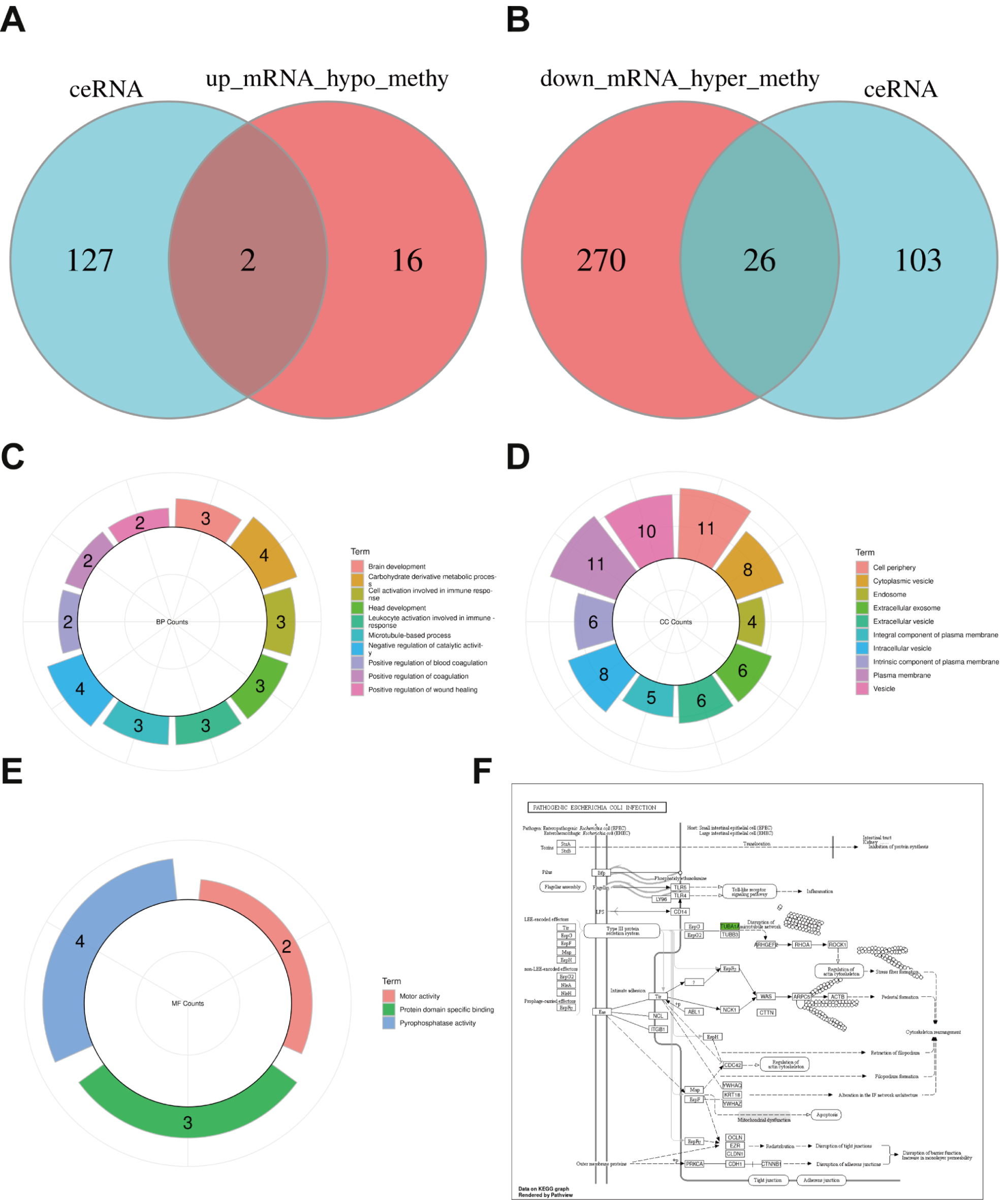

Supplement: Supplementary file 4 — Supplementary Material 4. S Fig. 4. Analysis of dysregulated genes shared by ceRNA regulation and DNA methylation in nasopharyngeal carcinoma. (A) Venn diagram of the up-regulated genes regulated by ceRNA and hypomethylation. (B) Venn diagram of the down-regulated genes regulated by ceRNA and hypermethylation. (C−E) GO enrichment results of the down-regulated genes regulated by ceRNA and hypermethylation, including (C) biological processes, (D) cellular component, and (E) molecular function. (F) Pathogenic Escherichia coli infection pathway enriched by down-regulated genes is regulated by ceRNA and hypermethylation [file 12920_2023_1653_MOESM4_ESM.tif]

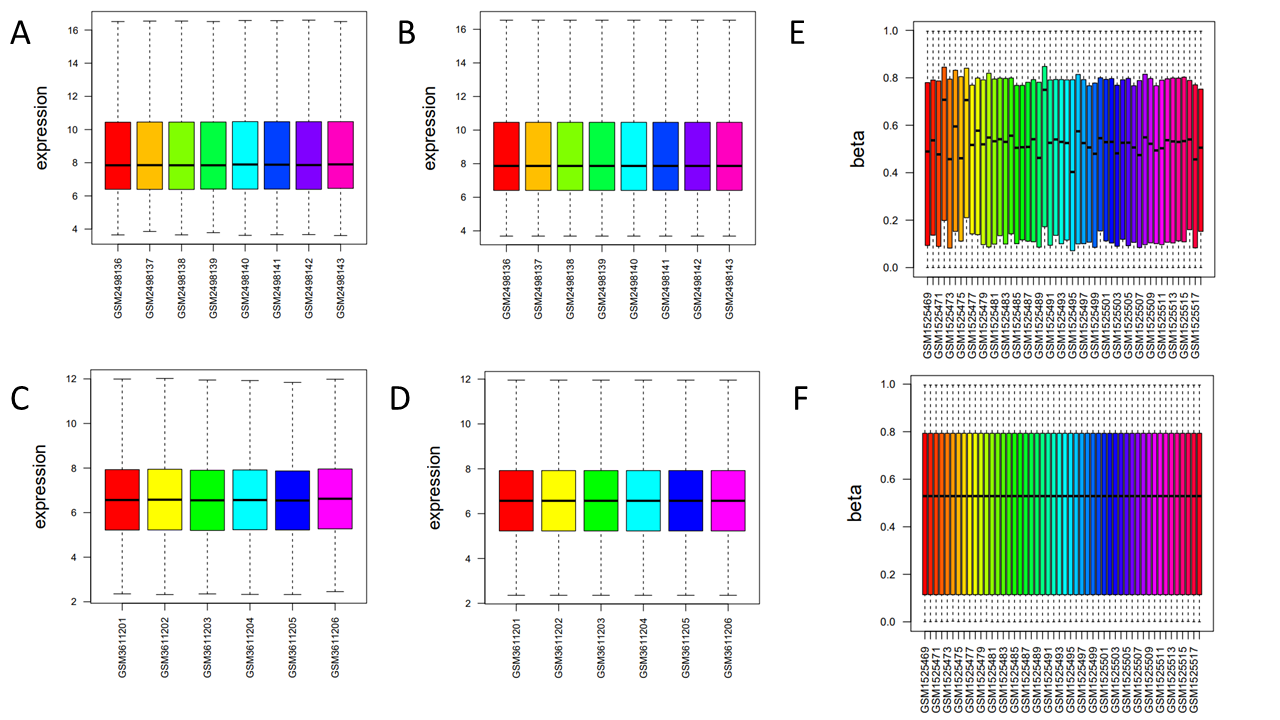

Supplement: Supplementary file 5 — Supplementary Material 5. S Fig. 5. Before and after comparison of lncRNAGSE95166 database and GSE126683 database, and quality control standards of methylation data. (A) lncRNA database GSE95166 before standardization (B) lncRNA database GSE95166 after standardization. (C) Before lncRNA GSE126683 standardization. (D) After lncRNA GSE126683 standardization. (E) Before the standardization of methylation data. (F) After the standardization of methylation data [file 12920_2023_1653_MOESM5_ESM.tif]

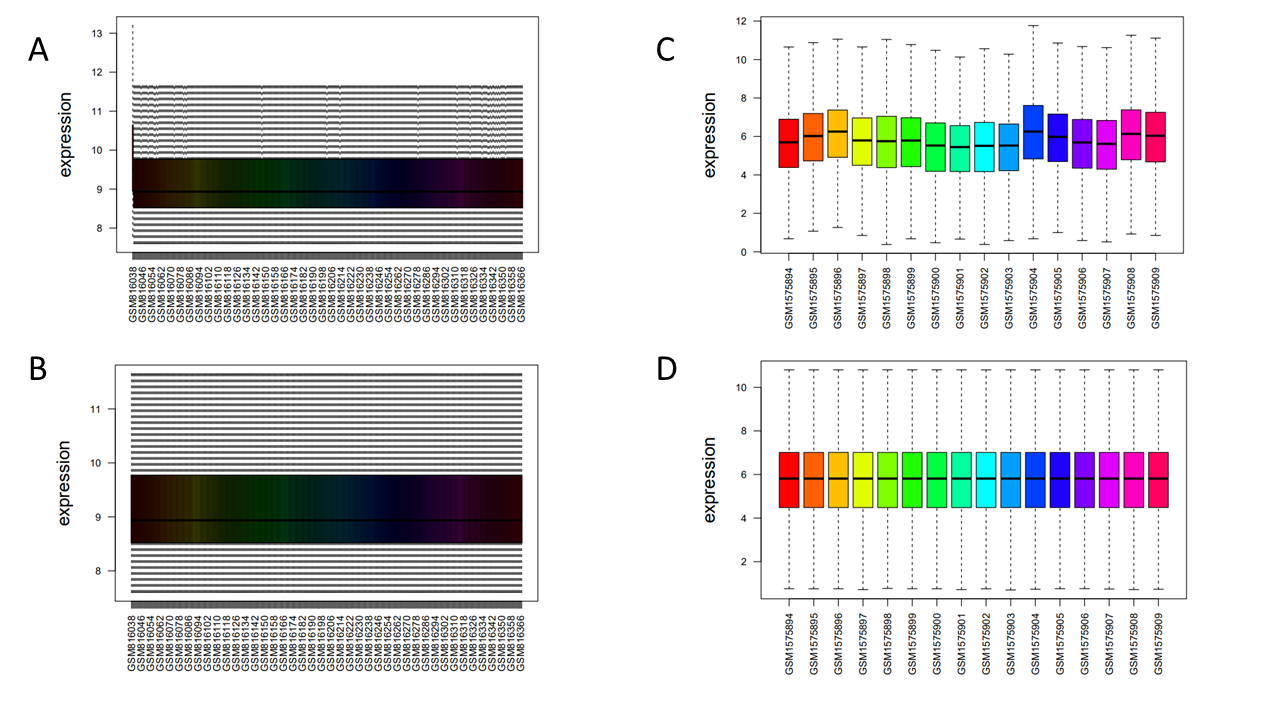

Supplement: Supplementary file 6 — Supplementary Material 6. S Fig. 6. Comparison of quality control standards before and after miRNA database and mRNA database. (A) Before miRNA database standardization. (B)miRNA database after standardization.(C) Before the standardization of mRNA database. (D) After the standardization of mRNA database [file 12920_2023_1653_MOESM6_ESM.tif]

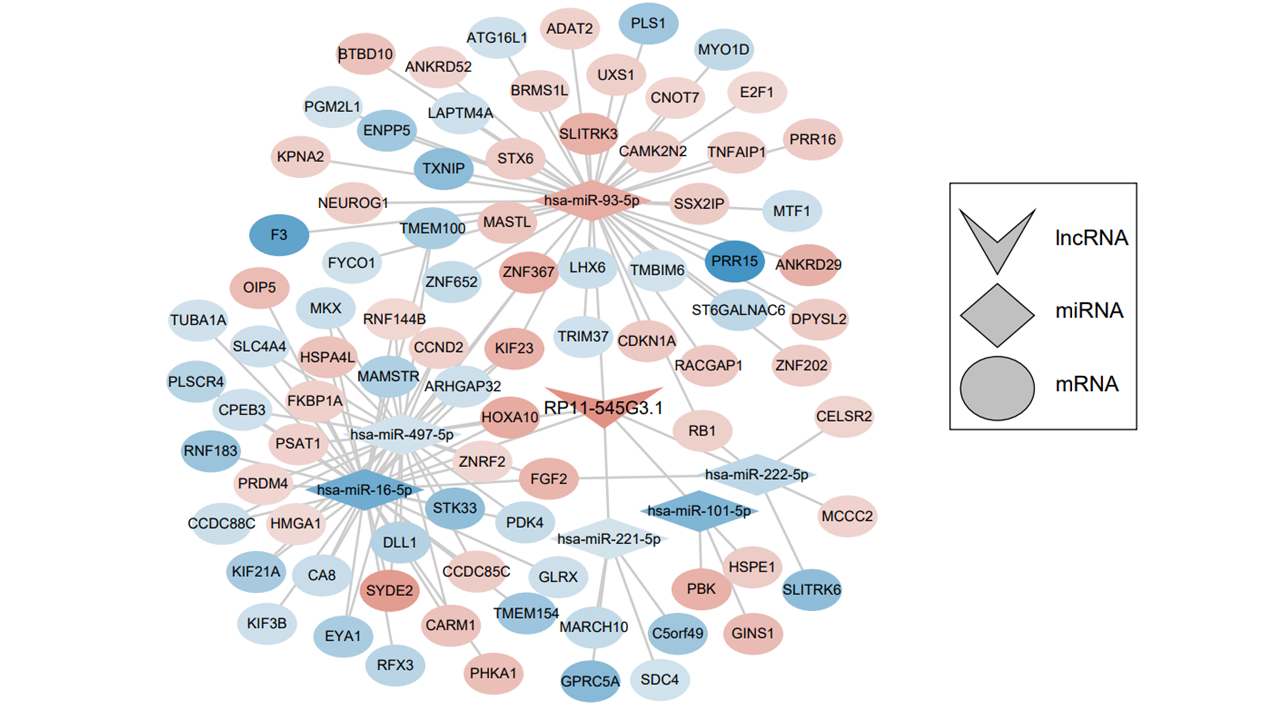

Supplement: Supplementary file 7 — Supplementary Material 7. S Fig. 7. Regulatory network of miRNAs and mRNAs related to RP11-545G3.1. [file 12920_2023_1653_MOESM7_ESM.tif]
