# Supplementary material for: Uncovering the ceRNA network and DNA methylation associated with gene expression in nasopharyngeal carcinoma
Source: BMC Med Genomics. 2023 Sep 14;16:218. doi: 10.1186/s12920-023-01653-1 (PMC10500855; doi:10.1186/s12920-023-01653-1)
Supplement: Supplementary file 8 — Supplementary Material 8 [file 12920_2023_1653_MOESM8_ESM.pptx]

## Slide 1
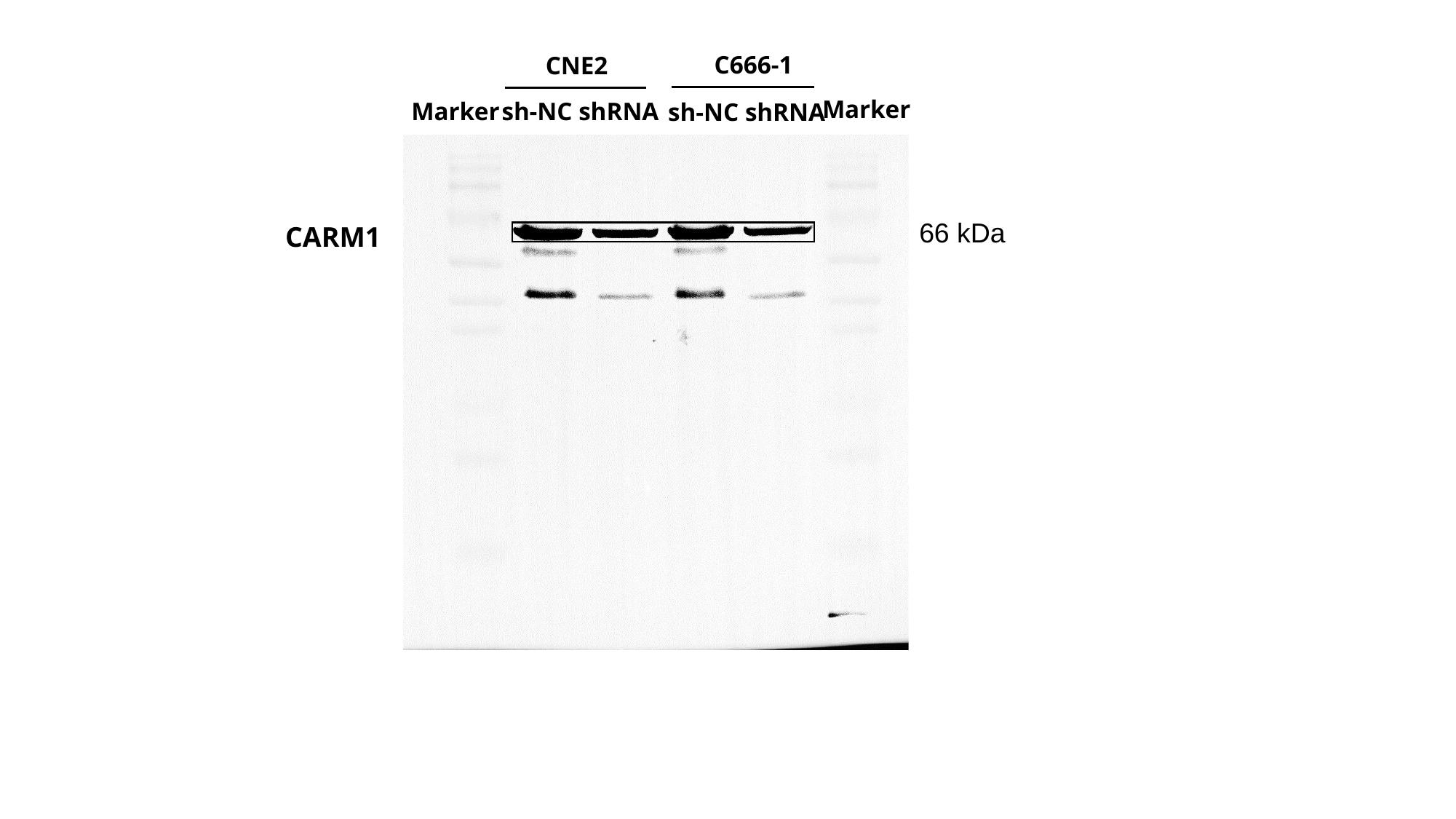

C666-1
CNE2
Marker
Marker
sh-NC shRNA
sh-NC shRNA
66 kDa
CARM1

## Slide 2
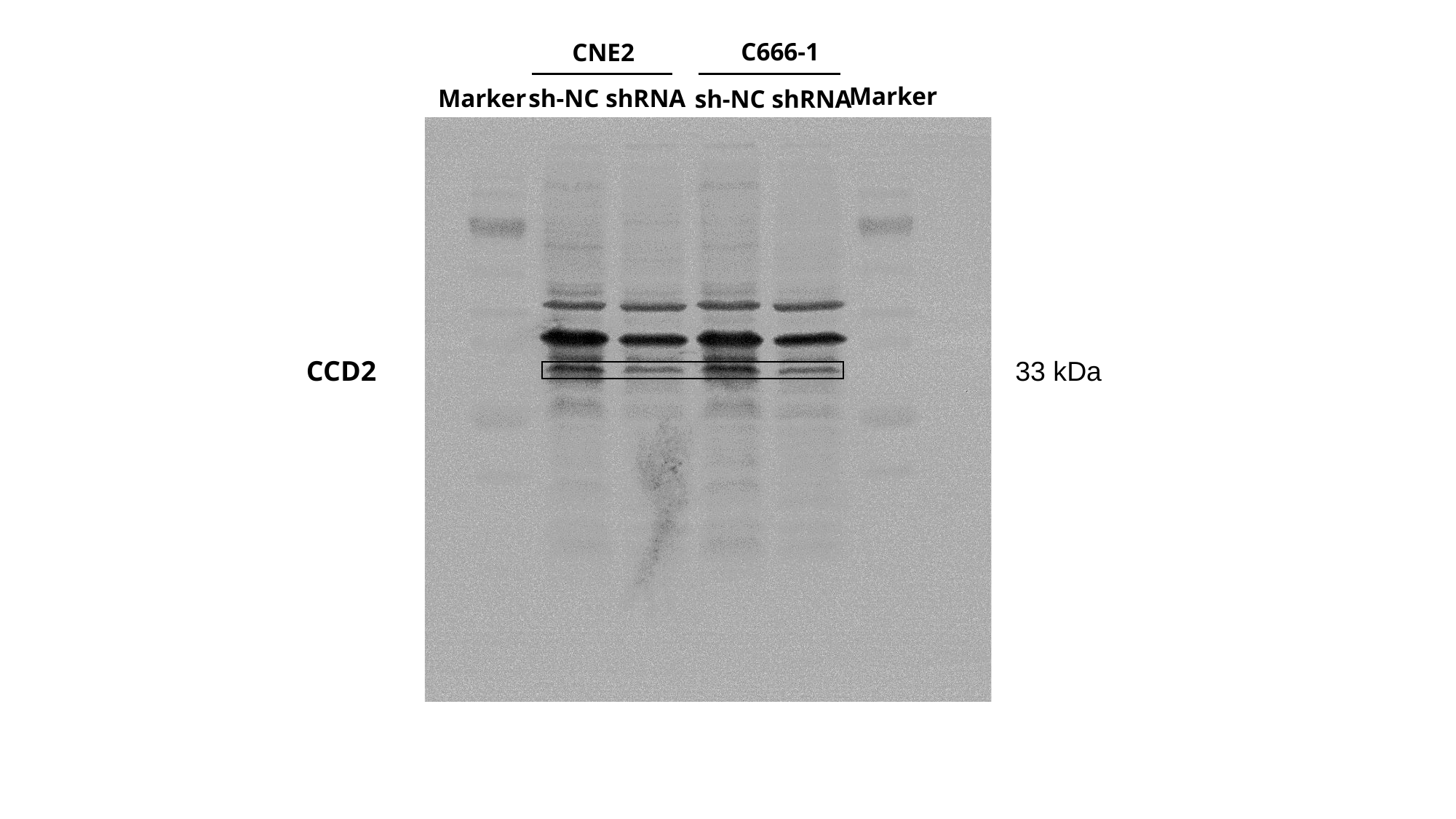

C666-1
CNE2
Marker
Marker
sh-NC shRNA
sh-NC shRNA
CCD2
33 kDa

## Slide 3
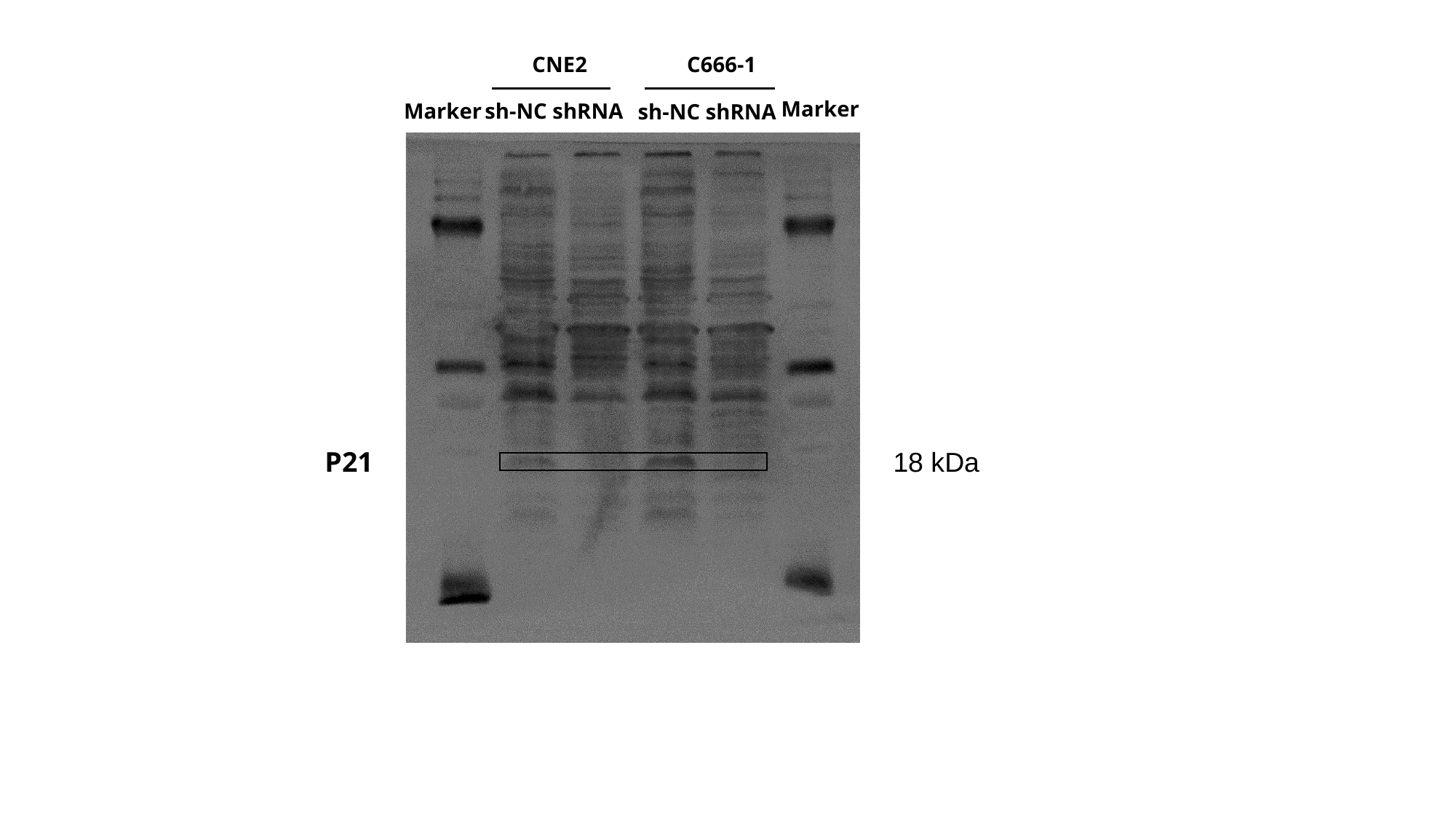

C666-1
CNE2
Marker
Marker
sh-NC shRNA
sh-NC shRNA
P21
18 kDa

## Slide 4
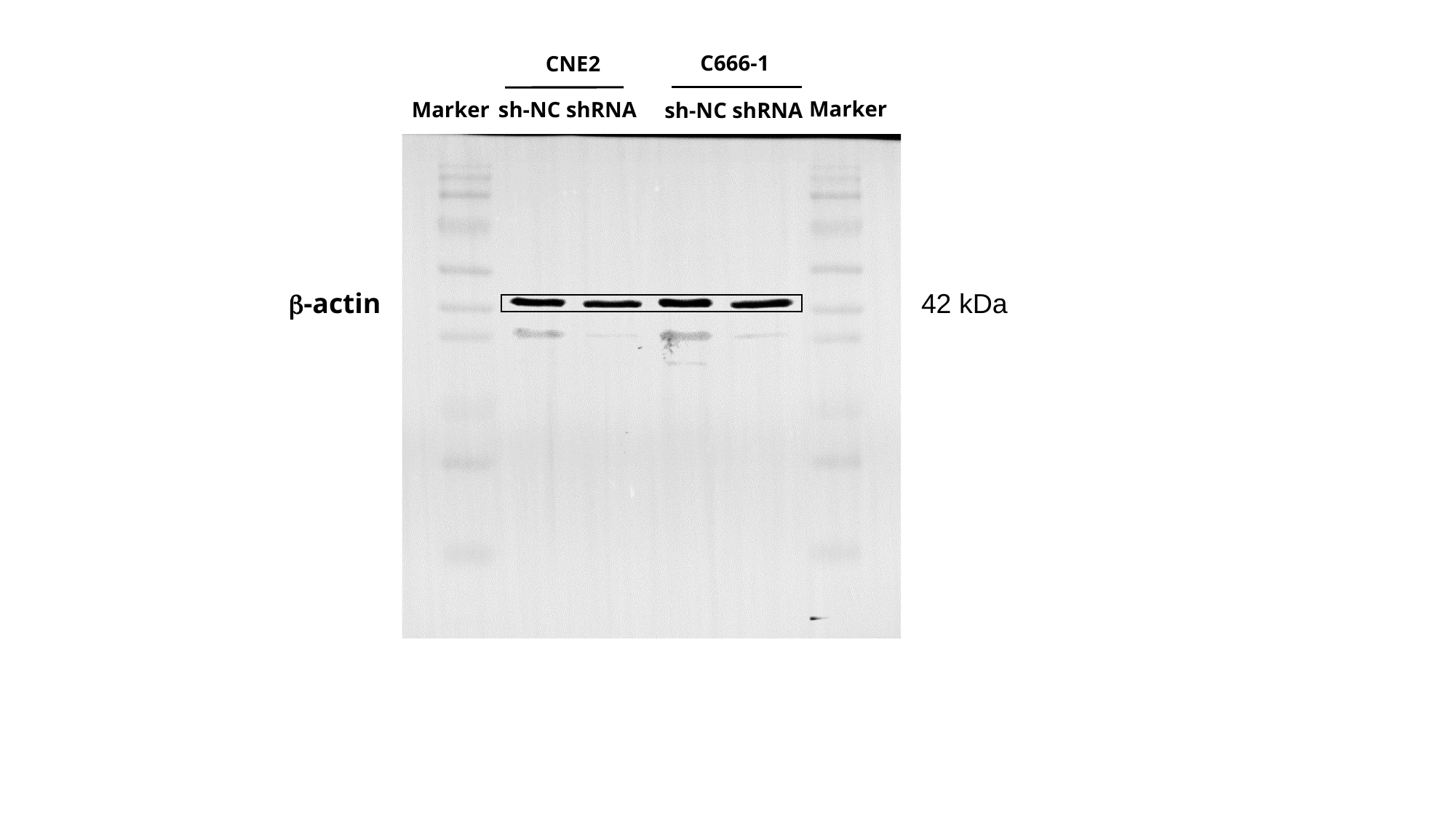

C666-1
CNE2
Marker
Marker
sh-NC shRNA
sh-NC shRNA
b-actin
42 kDa
