# Supplementary material for: Uncovering the ceRNA network and DNA methylation associated with gene expression in nasopharyngeal carcinoma
Source: BMC Med Genomics. 2023 Sep 14;16:218. doi: 10.1186/s12920-023-01653-1 (PMC10500855; doi:10.1186/s12920-023-01653-1)
Supplement: Supplementary file 9 — Supplementary Material 9 [file 12920_2023_1653_MOESM9_ESM.docx]

**Uncovering the** **ceRNA network and DNA methylation associated with gene expression in nasopharyngeal carcinoma**

Ting Zhang^1*^ , Lu Pei^2*^, Wen-Li Qiu^2^, Yu-xia Wei ^1^ , Bi-yun Liao^1^, Feng-lian Yang^2#^

^1^ Center of Reproductive medicine, Affiliated hospital of Youjiang Medical University for Nationalities, Baise, Guangxi, China, 533000

^2^ Youjiang medical university for nationalities, Baise, Guangxi, China, 533000

**Running title:** ceRNA network, DNA methylationin nasopharyngeal carcinoma

* These two authors contributed equally to this work.

**^#^Corresponding author:** Feng-lian Yang

**Email:** Yangfenglian303@126.com

**Abstract**

**Objective:** This study aimed to uncover abnormally expressed genes regulated by competitive endogenous RNA (ceRNA) and DNA methylation nasopharyngeal carcinoma and to validate the role of lncRNAs in the ceRNA network on nasopharyngeal carcinoma progression.

**Methods:** Based on the GSE64634 (mRNA), GSE32960 (miRNA), GSE95166 (lncRNA), and GSE126683 (lncRNA) datasets, we screened differentially expressed mRNAs, miRNAs and lncRNAs in nasopharyngeal carcinoma. A ceRNA network was subsequently constructed. Differentially methylated genes were screened using the GSE62336 dataset. The abnormally expressed genes regulated by both the ceRNA network and DNA methylation were identified. In the ceRNA network, the expression of RP11-545G3.1 lncRNA was validated in nasopharyngeal carcinoma tissues and cells by RT-qPCR. After a knockdown of RP11-545G3.1, the viability, migration, and invasion of CNE-2 and NP69 cells was assessed by CCK-8, wound healing and Transwell assays.

**Results:** This study identified abnormally expressed mRNAs, miRNAs and lncRNAs in nasopharyngeal carcinoma tissues. A ceRNA network was constructed, which contained three lncRNAs, 15 miRNAs and 129 mRNAs. Among the nodes in the PPI network based on the mRNAs in the ceRNA network, HMGA1 was assessed in relation to the overall and disease-free survival of nasopharyngeal carcinoma. We screened two up-regulated genes regulated by the ceRNA network and hypomethylation and 26 down-regulated genes regulated by the ceRNA network and hypermethylation. RP11-545G3.1 was highly expressed in the nasopharyngeal carcinoma tissues and cells. Moreover, the knockdown of RP11-545G3.1 reduced the viability, migration, and invasion of CNE-2 and NP69 cells.

**Conclusion:** Our findings uncovered the epigenetic regulation in nasopharyngeal carcinoma and identified the implications of RP11-545G3.1 on the progression of nasopharyngeal carcinoma.

**Keywords:** nasopharyngeal carcinoma; competitive endogenous RNA; DNA methylation; epigenetic regulation; RP11-545G3.1

**Introduction**

Nasopharyngeal carcinoma is a malignant tumor that occurs in the nasopharyngeal mucosa epithelium and small salivary gland, which is primarily a poorly differentiated squamous cell carcinoma [1]. Nasopharyngeal carcinoma is highly malignant and prone to metastasis, as well as recurrence. This malignancy is especially prevalent in the regions of east and southeast Asia. Unfortunately, approximately 20% of patients with nasopharyngeal carcinoma continue to have local recurrence and distant metastasis after treatment, which is mainly due to the radioresistance of nasopharyngeal carcinoma [2][3]. Because nasopharyngeal carcinoma is highly malignant and progresses rapidly, most patients are diagnosed as the middle and advanced stages. The 5-year survival rate of patients with advanced nasopharyngeal carcinoma is less than 60% [3]. Therefore, it is significant to investigate further treatment strategies to provide novel options for treating nasopharyngeal carcinoma.

The analysis of molecular characteristics using high-throughput sequencing has greatly improved our understanding of the pathogenesis of nasopharyngeal carcinoma. Non-coding RNAs (e.g., long-chain non-coding RNAs [lncRNAs] and microRNAs [miRNAs]) are involved in regulating physiological processes and pathogenesis of various diseases [4]. Dysregulated competing endogenous RNA (ceRNA) is related to the occurrence and progression of cancers, including nasopharyngeal carcinoma [5]. CeRNA acts as a sponge of miRNA via an miRNA response element, thereby forming an intricate ceRNA network [6]. Nevertheless, the molecular mechanisms of ceRNA have not been fully elucidated in nasopharyngeal carcinoma. The pathogenesis of nasopharyngeal carcinoma is a biological process which includes both genetic and environmental factors. Moreover, the accumulation of genomic and epigenetic changes contributes to the occurrence of nasopharyngeal carcinoma [7]. DNA methylation is the most common modification in epigenetics, and its abnormal changes represent the main factors that lead to the development of various tumors, including nasopharyngeal carcinoma [8]. Abnormal methylation can affect the function of key genes by changing their expression and thus affecting various processes during tumor development [9]. Gene-specific changes in methylation that occur during the early stages of cancer can assist with early cancer detection and prevention [10]. Thus, it is important to comprehensively uncover abnormally expressed genes that are regulated by DNA methylation.

This study aimed to uncover the ceRNA network and DNA methylation associated with gene expression in nasopharyngeal carcinoma and validate the function of RP11-545G3.1 lncRNA on the progression of nasopharyngeal carcinoma.

**Materials and methods**

**Nasopharyngeal carcinoma and preprocessing datasets**

The mRNA, miRNA and lncRNA expression profiles and DNA methylation profiles of nasopharyngeal carcinoma samples were retrieved from the Gene Expression Omnibus (GEO; <https://www.ncbi.nlm.nih.gov/gds/>) database. The mRNA expression profiles of 12 nasopharyngeal carcinoma and four normal nasopharyngeal samples on the GPL570 platform were extracted from the GSE64634 dataset [11]. The miRNA expression profiles of 312 paraffin-embedded nasopharyngeal carcinoma and 18 normal nasopharyngeal tissues were obtained from the GSE32960 dataset on the GPL14722 platform [12]. Furthermore, the lncRNA expression profiles of four nasopharyngeal carcinoma and para-carcinoma tissues were retrieved from the GSE95166 dataset using the GPL15314 platform [13]. In addition, this study downloaded the lncRNA expression profiles of three paired nasopharyngeal carcinoma and normal nasopharynx specimens on the GPL16956 platform [5]. The expression matrix data were normalized by quartile utilizing the normalizeBetweenArrays option in the limma package [14]. Moreover, the DNA methylation data were normalized using the wateRmelon package[15]. Outliers were detected and removed by employing a principal component analysis (PCA) using the psych package. The correlation between samples at the gene expression level was analyzed and visualized as heat maps. R v3.6.0 were applied in this study with R packages including limma v3.1.6, Cytoscape v3.7, STRING v11, survival v2.43 were applied in this study[14, 16, 17].

**Differential analysis**

Differentially expressed mRNAs, miRNAs and lncRNAs, as well as differentially methylated genes (DMGs) were screened between nasopharyngeal carcinoma and normal nasopharyngeal tissue samples by applying the limma package (version 3.5.1) [18](**S Figure 1**). The screening threshold was set as |fold change (FC)| ≥ 1.5 and adjusted *P* < 0.05. The results were visualized into a volcano map and clustering heatmap using the heatmap.2 package [19]. Quality control (QC) of raw sequences ensures the availability of RNA-seq data and reduces biases introduced by sequencing depth, read distribution, and coverage uniformity, etc. Supplementary plots on the possible batch effects and quality control of each of the analyzed datasets should be included. (**S Figure 5-6**).

**Function enrichment analysis**

Based on the Gene Ontology (GO) database [20] and the Kyoto Encyclopedia of Genes and Genomes (KEGG) Pathway database [21], the functional enrichment of differentially expressed genes (DEGs) or DMGs was analyzed. A Fisher’s exact test was used to identify which group of genes exhibited the highest association with specific functional items. The P-value and false discovery rate (FDR) corresponding to each item was calculated [22]. Items with FDR < 0.05 were considered to be significantly enriched.

**Construction of a ceRNA network**

Target mRNAs of differentially expressed miRNAs were predicted by an integration of the miRTarBase (http://miRTarBase.cuhk.edu.cn/) [23], TargetScan (targetscan.org) [24], miRDB (http://mirdb.org) [25], miRanda (miranda.org/) and miRMap (mirnamap.mbc.nctu.edu.tw/) databases. Furthermore, miRNA-lncRNA relationships were predicted by implementing the miRcode database (http://www.mircode.org) [26]. The miRNA-mRNA and miRNA-lncRNA interactions were imported into Cytoscape software to construct a ceRNA network [27]. Furthermore, a GO and KEGG enrichment analysis of mRNAs in the ceRNA network was presented using the Cytoscape plugin, ClueGO [28].

**Protein-protein interaction (PPI) network**

The mRNAs in the ceRNA network were analyzed with the STRING online tool (http://www.bork.embl-heidelberg.de/STRING/) [29]. The required confidence (combined score) was set as > 0.7. The PPI network was visualized via Cytoscape [30].

**Patients and specimens**

This study included 21 patients with nasopharyngeal carcinoma admitted to the Affiliated Hospital of Youjiang Medical College for Nationalities from January 2018 to January 2019. Samples of nasopharyngeal carcinoma and matched normal tissues were collected from each patient. All patients underwent a pathological biopsy, and did not receive radiotherapy or chemotherapy prior to the biopsy. The biopsy combined with clinical manifestations and CT examination of the nasopharynx were used for a diagnosis of nasopharyngeal squamous cell carcinoma. The following exclusion criteria were used: 1) age < 18 or > 75 years; 2) combined with other primary tumors or metastatic cancer; 3) severe infectious disease and immune dysfunction; 4) pregnant and lactating women; and 5) mental disorders. All specimens were stored in liquid nitrogen. This study was approved by the Ethics Committee of Affiliated Hospital of Youjiang Medical College for Nationalities, Guangxi province, China, and all patients provided informed consent.

**RT-qPCR**

The total RNA from tumor samples was extracted using the TRIzol method (Roche, Mannheim, Germany). The extracted RNA was reverse transcribed into cDNA using a reverse transcription kit (Roche, Mannheim, Germany). A Roche Lightcycler480 RT-qPCR instrument (Roche, Mannheim, Germany) was used for amplification. The reaction conditions were as follows: pre-denaturation at 95°C for 10 min, denaturation at 95°C for 15 s and annealing at 60°C for 1 min, for a total of 40 cycles, followed by extension at 72°C for 10 min. The β-actin was used as an internal control to normalize the RT-qPCR results. Primer-BLAST was employed to design the primers, and specific primer sequences were synthesized by Shanghai Shenggong Biological Engineering Co., Ltd. (China). The following primer sequences were used in this study: RP11-545G3.1, 5´-GGACAAACCCTACTGTGA-3´ (forward), 5´-CCCTGGACGTGACTTCTT-3´ (reverse) and β-actin, 5´-CCTAGAAGCATTTGCGGTGG-3´ (forward), 5´-GAGCTACGAGCTGCCTGACG-3´ (reverse).

**Cell culture**

Human nasal mucosa epithelial cells (HNMEpiC; P10567; Beijing Bright Biotechnology Co., Ltd., China) and human nasopharyngeal carcinoma cells CNE-1 (CC-Y1118; EK-Bioscience), CNE-2 (CC-Y1119; EK-Bioscience), C666-1 (CC-Y1082; EK-Bioscience) and NP69 (BFN60870097; Shanghai Cell Bank, China) were cultured in RPMI-1640 (Gibco; Grand Island, NY, USA) containing 10% fetal bovine serum and 1% P/S at 37℃ and 5% CO_2_. The cells were trypsinized and passaged once the cells had adhered to the wall of a 96-well plate (Corning, UK) and grown to a confluence of over 90%.

**Transfection**

CNE-2 and C666-1 cells were seeded into a 6-well plate (2 × 10^5^ cells/well). After culturing the cells in antibiotic-free cell culture medium for 24 h, the cell fusion rate reached 80%~85%. A Lipofectamine^™^2000 transfection kit (Invitrogen; USA) was performed in accordance with the manufacturer’s instructions, and shRNAs against RP11-545G3.1 (GenePharma, Shanghai, China) and sh-negative control were transfected into the cells. Fresh medium was applied 24 h after transfection and the cells were cultured for an additional 48 h. RT-qPCR was employed to detect the silencing effect of RP11-545G3.1.

**Cell counting kit-8 (CCK-8)**

The cells were seeded into a 96-well culture plate, and the cell density was adjusted to 5 × 10^3^ cells/well. Then, 10 µL CCK-8 solution (Beyotime, Shanghai, China) was added to each well and the cells were incubated for 4 h at 37°C before removing the supernatant. The samples were then incubated in 150 µL of a dimethyl sulfoxide solution for 10 min at 37°C. The absorbance value (OD value) at a wavelength of 450 nm of the cells in each well was determined using a microplate reader (GENESYS 10S UV-Vis, Thermo Fisher Scientific, UK).

**Western blot**

The total protein from the tumor samples or cells was extracted using a cell protein extraction kit (Thermo, USA). The bicinchoninic acid (Zhongshan Jinqiao Biotechnology Co., Ltd., Beijing, China) method was used to determine the total protein concentration and to perform a quantitative detection of the samples. A sodium lauryl sulfate-polyacrylamide gel was prepared, and 50 µg of protein sample was added to each well. After subjecting the samples to 75 V constant voltage electrophoresis for 30 min, 100 V constant voltage electrophoresis was performed for 2 h. The wet transfer method was applied to electrically transfer the protein to a polyvinylidene fluoride membrane. After blocking the membrane with 5% bovine serum albumin for 60 min, the corresponding primary antibody was added and incubated overnight at 4°C. Next, a secondary antibody (goat anti-rabbit IgG H&L (HRP) preabsorbed, 1/1000; ab7090; Abcam, Cambridge, MA, USA) was incubated with the membrane for 1 h at room temperature. Using an Odyssey two-color infrared laser imaging system, the membrane was scanned. β-actin was used as an internal control to analyze the level of target protein expression. The primary antibodies included CARM1 (1/5,000; ab245466; Abcam), CCND2 (1/500; ab230883; Abcam), P21 (1/500; ab227443; Abcam) and β-actin (1/5,000; ab179467; Abcam).

**Wound healing assay**

The cells were seeded in a 12-well plate (4 × 10^4^/well). After 48 h, a 10 μL pipette tip was used to create a mark on the horizontal line perpendicular to the back of the culture plate. Serum-free culture medium was added and the cells were continued culturing for 24 h. At 0 h and 24 h, three fields of view were randomly selected for each well under a fluorescence microscope.

**Transwell assay**

Matrigel (BD Biosciences; San Jose, CA, USA) was spread evenly over the bottom of the upper Transwell chamber (Corning; NY, USA). After the Matrigel was completely solidified, the remaining liquid in the upper chamber was removed, and 50 μL of serum-free DMEM medium was added. The shRNA-treated cells were resuspended into 200 μL serum-free medium and inoculated into the upper layer of the chamber (4 × 10^4^ cells/well). At the same time, 500 μL cell culture medium containing 20% FBS was added to the lower Transwell chamber. After the Transwell chamber was incubated in a cell incubator for 24 h, the medium was removed and the cells in the upper chamber were removed with a cotton swab and then fixed in 10% methanol solution for 20 min. After rinsing the chambers three times with PBS, the cells were stained with crystal violet solution for 15 min. Three fields were randomly selected for high-power imaging.

**Statistical analysis**

Statistical analysis was performed using R language and GraphPad Prism software (version 8.0, San Diego, CA, USA, www.graphpad.com). The measurement data were displayed as the mean ± standard deviation. Kaplan-Meier curves for the overall survival and disease-free survival of the genes with degree ≥ 2 in the PPI network were carried out for the TCGA-HNSC dataset utilizing the “Survival” package, followed by a log-rank test. A Student’s *t*-test was applied to compare the differences between two groups. Multiple comparisons were analyzed by a one-way ANOVA, followed by Tukey’s multiple comparisons test. *P* < 0.05 was considered to be statistically significant.

**Results**

**Analysis of differentially expressed mRNAs in** **nasopharyngeal carcinoma**

The mRNA expression profiles of 12 nasopharyngeal carcinoma and 4 normal samples were extracted from the GSE64634 dataset. The PCA results revealed that nasopharyngeal carcinoma samples differed from normal samples (**Figure 1A**). There was a high correlation between the samples at the mRNA level (**Figure 1B**). Compared with normal samples, there were 1,078 up-regulated and 1,113 down-regulated mRNAs in the nasopharyngeal carcinoma samples (**Figure 1C−E**). The results of the GO enrichment analysis showed that these up-regulated mRNAs were primarily related to cellular proliferation (e.g., cell cycle, mitotic cell cycle, DNA replication, nuclear DNA replication, DNA-dependent DNA replication, cell division, chromosome segregation, mitotic nuclear division and mitotic cell cycle phase transition) (**Figure 1F**). The down-regulated mRNAs were primarily correlated with cell motility (**Figure 1G**). Similarly, the up-regulated mRNAs were mainly enriched in cell proliferation-related pathways (**Figure 1H**) whereas the down-regulated mRNAs were enriched in relation to metabolic pathways (**Figure 1I**).

**Analysis of abnormally expressed miRNAs in nasopharyngeal carcinoma**

We obtained the miRNA expression profiles of 312 nasopharyngeal carcinoma and 18 normal specimens from the GSE32960 dataset. According to the PCA results, no outlier samples were removed (**S** **Figure 2A**). A high correlation between the samples was detected, as shown in **S Figure 2B**. There were 91 up- and 94 down-regulated miRNAs in the nasopharyngeal carcinoma specimens in comparison to that of the normal specimens (**S Figure 2C−E**). Based on intersections of the target mRNAs of differentially expressed miRNAs from miRTarBase, TargetScan, miRDB, miRanda and miRMap databases, 220 mRNAs were identified to be targeted by these miRNAs (**S** **Figure 2F**).

**Screening of dysregulated lncRNAs in nasopharyngeal carcinoma**

Nasopharyngeal carcinoma-related lncRNAs were analyzed using the GSE95166 and GSE126683 datasets. Nasopharyngeal carcinoma samples were distinctly differentiated from the normal samples in both the GSE95166 (**Figure 2A**) and GSE126683 datasets (**Figure 2B**). In addition, a relatively high correlation was observed between them in the two datasets (**Figure 2C and D**). A total of 2,307 up- and 2,356 down-regulated lncRNAs were detected in nasopharyngeal carcinoma in the GSE95166 dataset, whereas 314 up- and 577 down-regulated lnRNAs were identified in nasopharyngeal carcinoma in the GSE126683 dataset (**Figure 2E−J**). Following the intersection of the results from the two datasets, we obtained five up-regulated lncRNAs (LOC100144603, RP11-545G3.1, SCARNA22, TAZ and TIMM8B; **Figure 2K**) and six down-regulated lncRNAs (AC003986.7, AK055386, BC013821, DQ786304, LOC647979 and RP11-179H18.2; **Figure 2L**) in nasopharyngeal carcinoma.

**Construction of a ceRNA network in nasopharyngeal carcinoma**

After matching the dysregulated miRNA-mRNA and lncRNA-miRNA pairs, a ceRNA network was established for nasopharyngeal carcinoma (**Figure 3A**). The network was comprised of three lncRNAs (RP11-179H18.2, RP11-545G3.1 and TAZ), 15 miRNAs (hsa-miR-101-5p, hsa-miR-125a-5p, hsa-miR-125b-5p, hsa-miR-142-3p, hsa-miR-143-5p, hsa-miR-145-5p, hsa-miR-150-5p, hsa-miR-16-5p, hsa-miR-205-5p, hsa-miR-21-5p, hsa-miR-221-5p, hsa-miR-222-5p, hsa-miR-425-5p, hsa-miR-497-5p and hsa-miR-93-5p), and 129 mRNAs. We further probed into the functions of the mRNAs in the ceRNA network. **Figure 3B** shows that these mRNAs were mainly enriched in cancer-related pathways. Furthermore, the mRNAs were primarily related to DNA integrity checkpoints and DNA damage checkpoints (**Figure 3C**). In addition, they participated in regulating cellular components of cyclin-dependent protein kinase holoenzyme complex, kinesin complex, and Flemming bodies (**Figure 3D**). **Figure 3E** demonstrated that the 129 mRNAs in the ceRNA network had the molecular functions of RNA polymerase II transcription factor binding, histone deacetylase binding, protein phosphatase 2A binding, protein kinase C binding and protein self-association.

**Establishment of a PPI network based on mRNAs in the ceRNA network in nasopharyngeal carcinoma**

We established a PPI network containing 38 nodes in nasopharyngeal carcinoma based on the mRNAs in the ceRNA network using the STRING database (**Figure 4A**). There was a total of 29 up-regulated and nine down-regulated mRNAs in the nasopharyngeal carcinoma samples compared to that of the normal specimens. **Table 1** lists the degree of nodes in the PPI network, among which, TP53 had the highest degree (n = 13). The functions of the nodes in the network were further analyzed. **Figure 4B** shows that the cell cycle and metabolic processes were significantly enriched. These genes were found to be involved in key cellular components like the chromosomes, cytoplasm, and cytoskeleton (**Figure 4C**). In addition, the mRNAs in the PPI network had the complex molecular functions of adenyl nucleotide, ATP, and enzyme binding (**Figure 4D**). The KEGG enrichment results demonstrated that various cancer-associated pathways were distinctly enriched by these genes (**Figure 4E**).

**HMGA1 is closely related to the prognosis of nasopharyngeal carcinoma.**

The prognostic values of the mRNAs with a degree ≥ 2 in the PPI network were assessed in depth. As a result, only HMGA1 was found to be related to the prognosis of nasopharyngeal carcinoma. Patients with high HMGA1 expression were associated with undesirable overall survival (HR = 1.434; *P* = 8.63e^-03^; **Figure 5A**) and disease-free survival (HR = 1.466; *P* = 2.43e^-02^; **Figure 5B**) compared to those with low expression.

**Analysis of differentially methylated genes in nasopharyngeal carcinoma**

The GSE62336 dataset was employed to analyze DMGs in nasopharyngeal carcinoma. At the methylation level, distinct differences between nasopharyngeal carcinoma and normal samples were observed (**S** **Figure 3A**). A high correlation between the samples were found, as shown in **S Figure 3B**. A total of 12,383 hypermethylated and 853 hypomethylated sites were detected in the nasopharyngeal carcinoma specimens compared to that in the normal samples (**S** **Figure 3C−E**). To obtain the abnormally expressed genes that were affected by DNA methylation, we intersected DEGs with DMGs. As a result, 18 highly expressed and hypomethylated genes (**S** **Figure 3F**), as well as 296 lowly expressed and hypermethylated genes (**S** **Figure 3G**) were found in nasopharyngeal carcinoma. **S** **Figure 3H** shows the PPI network based on lowly expressed and hypermethylated genes. There were 84 nodes in the network, among which, DNAI2 and CCDC39 had the highest degree (n = 6). We also found that highly expressed and hypomethylated genes were distinctly enriched in metabolic and catabolic processes (**S** **Figure 3I**), as well as pyrimidine metabolism and small cell lung cancer pathways (**S** **Figure 3J**). Moreover, lowly expressed and hypermethylated genes were distinctly enriched in the processes governing cilium movement and assembly (S **Figure 3K**), as well as various metabolic and cancer-related pathways (**S** **Figure 3L**).

**Dysregulated genes shared by ceRNA regulation and DNA methylation in nasopharyngeal carcinoma**

We further analyzed the dysregulated genes shared by ceRNA regulation and DNA methylation in nasopharyngeal carcinoma. As a result, a total of two up-regulated genes (CTPS2 and SLITRK3) were found to be simultaneously regulated by ceRNA and hypomethylation (**S** **Figure 4A**). Furthermore, 26 down-regulated genes (*C5orf49*, *CA8*, *DLL1*, *ENPP4*, *F3*, *FYCO1*, *GPRC5A*, *HS3ST1*, *KIF21A*, *LAPTM4A*, *LHX6*, *MARCH10*, *MKX*, *MUC4*, *MYO1D*, *NFIA*, *PGM2L1*, *PLSCR4*, *PRR15*, *PTPRN2*, *RALGPS2*, *SLC4A4*, *STK33*, *TMEM154*, *TUBA1A and TXNIP*) were simultaneously regulated by ceRNA and hypermethylation (**S** **Figure 4B**). The down-regulated genes regulated by ceRNA and hypermethylation were significantly enriched in metabolic and immune response processes (**S** **Figure 4 C**) associated with various cellular components, such as the cell periphery, cytoplasmic vesicles, and endosomes (**S** **Figure 4D**). Furthermore, they exhibited molecular functions of motor activity, protein domain specific binding, and pyrophosphatase activity (**S** **Figure 4E**). **S** **Figure 4F** shows that the pathogenic *Escherichia coli* infection pathway was significantly enriched by these genes.

**LncRNA RP11-545G3.1 is up-regulated in nasopharyngeal carcinoma and its knockdown decreases nasopharyngeal carcinoma cell viability.**

Among the lncRNAs in the ceRNA network, RP11-545G3.1 was confirmed to be up-regulated in 21 paired nasopharyngeal carcinoma samples compared to that of normal samples (**Figure 6A**). Furthermore, we detected the expression of RP11-545G3.1 in different nasopharyngeal carcinoma cell lines. As a result, higher RP11-545G3.1 expression was detected in nasopharyngeal carcinoma cells (CNE-1, CNE-2, NP69 and C666-1) than in HNMEpiC cells (**Figure 6B**). To observe the functions of RP11-545G3.1 on nasopharyngeal carcinoma progression, RP11-545G3.1 was successfully silenced by shRNAs in both CNE-2 and NP69 cells (**Figure 6C**). The results of the CCK-8 assay showed that silencing RP11-545G3.1 distinctly lessened the cell viability of CNE-2 (**Figure 6D**) and NP69 cells (**Figure 6E**). Furthermore, we detected the expression of CARM1, CCND2 and P21 by performing a western blot (**Figure 6F**). A knockdown of RP11-545G3.1 distinctly reduced the expression of CARM1, CCND2 and P21 in both CNE-2 (**Figure 6G**) and NP69 cells (**Figure 6H**). We also conducted regulatory network of miRNAs and mRNAs related to RP11-545G3.1(**S** **Figure 7**).

**Knockdown of RP11-545G3.1 reduces the migration and invasion of nasopharyngeal carcinoma cells.**

The results of the wound healing assay revealed that a knockdown of RP11-545G3.1 distinctly reduced the migrated capacity of CNE-2 and NP69 cells (**Figure 7A, B**). Furthermore, we found that the invasive capacities of CNE-2 and NP69 cells were weakened by a RP11-545G3.1 knockdown (**Figure 7C and D**). Taken together, these findings indicated that silencing RP11-545G3.1 may decrease the migration and invasion of nasopharyngeal carcinoma cells.

**Discussion**

Nasopharyngeal carcinoma is the most common malignant tumor of the head and neck particularly in Southern China [3]. Because the anatomical components are hidden and difficult to detect early, most patients have already progressed to the middle and late stages of the disease at the time of diagnosis. And the prognosis of patients with advanced stage is not ideal due to the high degree of malignancy associated with nasopharyngeal carcinoma. Thus, clarification of the molecular mechanism associated with nasopharyngeal carcinoma progression will aid in the discovery of novel therapeutic targets and improve patient prognosis. With the continuous deepening of gene-targeted therapy research, the identification of effective target genes is of great significance for improving the prognosis of patients with nasopharyngeal carcinoma.

Furthermore, the specific genetic pathways involved in nasopharyngeal carcinoma remain elusive [31]. Microarray technology has assisted with probing into the various changes in genes in nasopharyngeal carcinoma, and has been confirmed as convenient method that can be used to screen for promising markers in nasopharyngeal carcinoma [13]. In this study, 1,078 up-regulated and 1,113 down-regulated mRNAs were identified in nasopharyngeal carcinoma tissues. These up-regulated mRNAs were primarily related to cellular proliferation whereas the down-regulated mRNAs were mainly correlated with cellular motility and metabolic pathways. Together, these findings indicated that these mRNAs may participate in the progression of nasopharyngeal carcinoma progression. As a ceRNA, lncRNA serves as an miRNA sponge, and may regulate the expression of tumor-related mRNAs. In the present study, a total of 91 up- and 94 down-regulated miRNAs were identified in nasopharyngeal carcinoma specimens. Furthermore, we screened five up-regulated lncRNAs (LOC100144603, RP11-545G3.1, SCARNA22, TAZ, and TIMM8B) and six down-regulated lncRNAs (AC003986.7, AK055386, BC013821, DQ786304, LOC647979 and RP11-179H18.2) in nasopharyngeal carcinoma specimens. It was previously found that SCARNA22 was overexpressed in breast cancer brain metastases [32]. However, the functions of other lncRNAs in cancer are poorly understood. We subsequently established a ceRNA network containing 3 lncRNAs (RP11-179H18.2, RP11-545G3.1, and TAZ), 15 miRNAs and 129 mRNAs. In previous studies, Zou et al. constructed a lncRNA-mediated ceRNA network for nasopharyngeal carcinoma using weighted correlation network analyses [33]. Moreover, Xu et al. identified the key genes related to a ceRNA network in nasopharyngeal carcinoma by applying bioinformatics analyses [13].

Based on the mRNAs in the ceRNA network, we established a PPI network containing 29 up- and nine down-regulated mRNAs in nasopharyngeal carcinoma. Among these mRNAs, only HMGA1 was found to be related to the overall survival and disease-free survival of nasopharyngeal carcinoma patients which will be further validated in a larger population. The carcinogenesis of HMGA1 has been confirmed in numerous malignant cancers [34]. For instance, HMGA1 may promote breast cancer aggressiveness and angiogenesis [35]. HMGA1 induces tumor growth via regulation of cell cycle as well as facilitates migrated and invasive capacities in cervical cancer [36]. Nevertheless, there is no research report on the role of HMGA1 in nasopharyngeal carcinoma.

Abnormal gene expression can be affected by DNA methylation [8]. We identified 18 highly expressed and hypomethylated genes, as well as 296 lowly expressed and hypermethylated genes in nasopharyngeal carcinoma specimens. We further analyzed two up-regulated genes (CTPS2 and SLITRK3) that were simultaneously regulated by ceRNA and hypomethylation and 26 down-regulated genes (C5orf49, CA8, DLL1, ENPP4, F3, FYCO1, GPRC5A, HS3ST1, KIF21A, LAPTM4A, LHX6, MARCH10, MKX, MUC4, MYO1D, NFIA, PGM2L1, PLSCR4, PRR15, PTPRN2, RALGPS2, SLC4A4, STK33, TMEM154, TUBA1A and TXNIP) that were simultaneously regulated by ceRNA and hypermethylation. Currently, the function of these mRNAs in nasopharyngeal carcinoma remains unclear. In the ceRNA network, LncRNA RP11-545G3.1 was verified to be up-regulated in nasopharyngeal carcinoma. A knockdown of RP11-545G3.1 reduced the level of cellular viability, migration, as well as nasopharyngeal carcinoma cell invasion. Further experiments should be performed to verify the functions of RP11-545G3.1 in this disease.

The advantage of this study is the comprehensive analysis from miRNA-mRNA and lncRNA-miRNA, the ceRNA networks related to nasopharyngeal carcinoma are gradually constructed. On the one hand, the interactions between RNA can be understood at the overall transcription level of cells. On the other hand, it can help to predict the target RNA and protein which may be related to the pathogenesis of nasopharyngeal carcinoma. Other highlights of this study include the first exposure of LncRNA RP11-545GG3.1, which is up-regulated in nasopharyngeal carcinoma and its knockdown decreases nasopharyngeal carcinoma cell viability. However, this study also has certain limitations: For the data used in this study are survival data of head and neck squamous cell carcinoma (and nasopharyngeal carcinoma belongs to head and neck squamous cell carcinoma), which may not represent the most real situation, but has certain clinical reference value. Another limitation of this paper is that batch effects or confounding factors within the data set were not adjusted for factors such as age in the EWAS study. In the future, we will expand the sample size of nasopharyngeal carcinoma and try to remove possible confounding factors for large-scale verification.

**Conclusion**

Collectively, this study revealed that the ceRNA network and abnormal DNA methylation were associated with gene expression in nasopharyngeal carcinoma, which deepened our understanding of the pathogenesis of this disease. Furthermore, we identified the novel lncRNA RP11-545G3.1 as a promising therapeutic target against nasopharyngeal carcinoma.

**Abbreviations:**

lncRNAs: long-chain non-coding RNAs; miRNAs: microRNAs; ceRNA: competing endogenous RNA; GEO: Gene Expression Omnibus; DMGs: differentially methylated genes; FC: fold change; DEGs: differentially expressed genes; FDR: false discovery rate; PPI: protein-protein interaction.

**Ethics approval and consent to participate**

Not applicable.

**Consent for publication**

Not applicable.

**Availability of data and material**

All the data were available by requesting to corresponding author F.L. Yang.

**Competing interests**

The authors declare no competing financial interests. All institutional and national guidelines for the care and use of laboratory animals were followed.

**Funding**

None.

**Authors' contributions**

T Zhang and L Pei performed the experiments, W.L. Qiu and Y.X. Wei analyzed the data, B.Y. Liao prepared the reagents, F.L. Yang designed the study and wrote the paper.

**Acknowledgements**

Not applicable.

**References:**

[1] EP H, WF L, BB M *et al.* Integrating post-radiotherapy plasma Epstein-Barr virus DNA and TNM stage for risk stratification of nasopharyngeal carcinoma to adjuvant therapy. 2020.

[2] Xiao WW, Huang SM, Han F *et al.* Local control, survival, and late toxicities of locally advanced nasopharyngeal carcinoma treated by simultaneous modulated accelerated radiotherapy combined with cisplatin concurrent chemotherapy: long-term results of a phase 2 study. Cancer 2011; 117:1874-1883.

[3] Chen YP, Chan ATC, Le QT *et al.* Nasopharyngeal carcinoma. Lancet 2019; 394:64-80.

[4] Beermann J, Piccoli MT, Viereck J, Thum T. Non-coding RNAs in Development and Disease: Background, Mechanisms, and Therapeutic Approaches. Physiol Rev 2016; 96:1297-1325.

[5] Zheng ZQ, Li ZX, Zhou GQ *et al.* Long Noncoding RNA FAM225A Promotes Nasopharyngeal Carcinoma Tumorigenesis and Metastasis by Acting as ceRNA to Sponge miR-590-3p/miR-1275 and Upregulate ITGB3. Cancer Res 2019; 79:4612-4626.

[6] Yin L, Chen J, Ma C *et al.* Hsa_circ_0046263 functions as a ceRNA to promote nasopharyngeal carcinoma progression by upregulating IGFBP3. Cell Death Dis 2020; 11:562.

[7] Baloche V, Ferrand FR, Makowska A *et al.* Emerging therapeutic targets for nasopharyngeal carcinoma: opportunities and challenges. Expert Opin Ther Targets 2020; 24:545-558.

[8] Lam WKJ, Jiang P, Chan KCA *et al.* Methylation analysis of plasma DNA informs etiologies of Epstein-Barr virus-associated diseases. Nat Commun 2019; 10:3256.

[9] Zheng XH, Wang RZ, Li XZ *et al.* Detection of methylation status of Epstein-Barr virus DNA C promoter in the diagnosis of nasopharyngeal carcinoma. Cancer Sci 2020; 111:592-600.

[10] Xu Y, Zhao W, Mo Y *et al.* Combination of RERG and ZNF671 methylation rates in circulating cell-free DNA: A novel biomarker for screening of nasopharyngeal carcinoma. Cancer Sci 2020; 111:2536-2545.

[11] Bo H, Gong Z, Zhang W *et al.* Upregulated long non-coding RNA AFAP1-AS1 expression is associated with progression and poor prognosis of nasopharyngeal carcinoma. Oncotarget 2015; 6:20404-20418.

[12] Zhang S, Yue W, Xie Y *et al.* The four‑microRNA signature identified by bioinformatics analysis predicts the prognosis of nasopharyngeal carcinoma patients. Oncol Rep 2019; 42:1767-1780.

[13] Xu Y, Huang X, Ye W *et al.* Comprehensive analysis of key genes associated with ceRNA networks in nasopharyngeal carcinoma based on bioinformatics analysis. Cancer Cell Int 2020; 20:408.

[14] Ritchie ME, Phipson B, Wu D *et al.* limma powers differential expression analyses for RNA-sequencing and microarray studies. Nucleic Acids Res 2015; 43:e47.

[15] Touleimat N, Tost J. Complete pipeline for Infinium(®) Human Methylation 450K BeadChip data processing using subset quantile normalization for accurate DNA methylation estimation. Epigenomics 2012; 4:325-341.

[16] Liu TH, Chen WH, Chen XD *et al.* Network Pharmacology Identifies the Mechanisms of Action of TaohongSiwu Decoction Against Essential Hypertension. Med Sci Monit 2020; 26:e920682.

[17] Szklarczyk D, Gable AL, Lyon D *et al.* STRING v11: protein-protein association networks with increased coverage, supporting functional discovery in genome-wide experimental datasets. Nucleic Acids Res 2019; 47:D607-d613.

[18] Ritchie ME, Belinda P, Di W *et al.* limma powers differential expression analyses for RNA-sequencing and microarray studies. 2015; 43:e47.

[19] Zhang H, Meltzer P, Davis SJBB. RCircos: an R package for Circos 2D track plots. 2013; 14:244.

[20] Ashburner M, Ball CA, Blake JA *et al.* Gene ontology: Tool for the unification of biology. 2000; 25:25-29.

[21] Minoru K, Susumu GJNAR. KEGG: Kyoto Encyclopedia of Genes and Genomes. 2000:1.

[22] Yoav, Benjamini, Yosef, B HJJotRSSS. Controlling the False Discovery Rate: A Practical and Powerful Approach to Multiple Testing. 1995.

[23] Huang HY, Lin YC, Li J *et al.* miRTarBase 2020: updates to the experimentally validated microRNA-target interaction database. Nucleic Acids Res 2020; 48:D148-d154.

[24] Agarwal V, Bell GW, Nam JW, Bartel DP. Predicting effective microRNA target sites in mammalian mRNAs. Elife 2015; 4.

[25] Chen Y, Wang X. miRDB: an online database for prediction of functional microRNA targets. Nucleic Acids Res 2020; 48:D127-d131.

[26] Jeggari A, Marks DS, Larsson E. miRcode: a map of putative microRNA target sites in the long non-coding transcriptome. Bioinformatics 2012; 28:2062-2063.

[27] Shannon P, Markiel A, Ozier O *et al.* Cytoscape: a software environment for integrated models of biomolecular interaction networks. Genome Res 2003; 13:2498-2504.

[28] Bindea G, Mlecnik B, Hackl H *et al.* ClueGO: a Cytoscape plug-in to decipher functionally grouped gene ontology and pathway annotation networks. Bioinformatics 2009; 25:1091-1093.

[29] Mikshowsky AA, Gianola D, Weigel KA. Assessing genomic prediction accuracy for Holstein sires using bootstrap aggregation sampling and leave-one-out cross validation. J Dairy Sci 2017; 100:453-464.

[30] Doncheva NT, Morris JH, Gorodkin J, Jensen LJ. Cytoscape StringApp: Network Analysis and Visualization of Proteomics Data. J Proteome Res 2019; 18:623-632.

[31] Chen Y, Zhou C, Li H *et al.* Identifying Key Genes for Nasopharyngeal Carcinoma by Prioritized Consensus Differentially Expressed Genes Caused by Aberrant Methylation. J Cancer 2021; 12:874-884.

[32] Schulten HJ, Bangash M, Karim S *et al.* Comprehensive molecular biomarker identification in breast cancer brain metastases. J Transl Med 2017; 15:269.

[33] Zou Z, Liu S, Ha Y, Huang B. Construction and Analysis of lncRNA-Mediated ceRNA Network in Nasopharyngeal Carcinoma Based on Weighted Correlation Network Analysis. Biomed Res Int 2020; 2020:1468980.

[34] Wang Y, Hu L, Zheng Y, Guo L. HMGA1 in cancer: Cancer classification by location. J Cell Mol Med 2019; 23:2293-2302.

[35] Zanin R, Pegoraro S, Ros G *et al.* HMGA1 promotes breast cancer angiogenesis supporting the stability, nuclear localization and transcriptional activity of FOXM1. J Exp Clin Cancer Res 2019; 38:313.

[36] Fu F, Wang T, Wu Z *et al.* HMGA1 exacerbates tumor growth through regulating the cell cycle and accelerates migration/invasion via targeting miR-221/222 in cervical cancer. Cell Death Dis 2018; 9:594.

**Figure legends:**

**Figure 1.** Analysis of differentially expressed mRNAs for nasopharyngeal carcinoma in the GSE64634 dataset. (A) PCA results of 12 nasopharyngeal carcinoma (green) and 4 normal samples (blue). (B) Heat map for the correlations between samples. The intensity of the color is proportional to the correlation coefficient. (C−E) Scatter plots, volcano diagram, and heat map of the differentially expressed mRNAs between nasopharyngeal carcinoma and normal samples. Blue: down-regulation; red: up-regulation. (F, G) The top 10 GO enrichment results of (F) up- and (G) down-regulated mRNAs. GO database: BP=biological process, CC=cellular component and molecular function, MT=molecular function. (H, I) The top 10 KEGG pathways enriched by (H) up- and (I) down-regulated mRNAs.

**Figure 2.** Screening of dysregulated lncRNAs in nasopharyngeal carcinoma in the GSE95166 and GSE126683 datasets. (A, B) PCA results of nasopharyngeal carcinoma and normal specimens. (C, D) Heat map for the correlations between samples. (E−J) Scatter plots, volcano diagram, and heat map for dysregulated lncRNAs between nasopharyngeal carcinoma and normal specimens. Blue: down-regulation; red: up-regulation. (K, L) Venn diagrams for the common (K) up- and (L) down-regulated lncRNAs in the GSE95166 and GSE126683 datasets.

**Figure 3.** Establishment of a nasopharyngeal carcinoma-related ceRNA network. (A) A ceRNA network containing lncRNAs (V type), miRNAs (diamond) and mRNAs (circle). The darker the color, the larger the |log_2_FC|. (B) KEGG enrichment results of the mRNAs in the ceRNA network. (C−E) GO enrichment results of the mRNAs in the ceRNA network, including (C) biological process, (D) cellular component, and (E) molecular function.

**Figure4.** Establishment of a PPI network based on the mRNAs in the ceRNA network for nasopharyngeal carcinoma. (A) A PPI network in nasopharyngeal carcinoma. Red: up-regulation; blue: down-regulation. The darker the color, the larger the |log2FC|. (B−D) GO enrichment results of mRNAs in the PPI network containing (B) biological processes, (C) cellular components, and (D) molecular functions. (E) KEGG enrichment pathways of mRNAs in the PPI network.

**Figure 5.** The relationship between HMGA1 expression and the prognosis of nasopharyngeal carcinoma. Kaplan-Meier curves of (A) overall survival and (B) disease-free survival between high and low expression HMGA1 groups.

**Figure 6.** Up-regulation of lncRNA RP11-545G3.1 in nasopharyngeal carcinoma and the effects of its knockdown on nasopharyngeal carcinoma cell viability. (A) RT-qPCR analysis of the expression of RP11-545G3.1 in 21 paired nasopharyngeal carcinoma and normal specimens. (B) RT-qPCR analysis of the expression of RP11-545G3.1 in nasopharyngeal carcinoma cells and normal cells. (C) Assessment of the silencing effects of shRNAs against RP11-545G3.1 in CNE-2 and NP69 cells by RT-qPCR. (D, E) CCK-8 assay of the cell viability of CNE-2 and NP69 cells transfected with shRNAs against RP11-545G3.1. (F−H) Western blot showing the expression of CARM1, CCND2 and P21 in CNE-2 and NP69 cells transfected with shRNAs against RP11-545G3.1. ^**^*P* < 0.01; ^***^*P* < 0.001; ^****^*P* < 0.0001.

**Figure 7.** A knockdown of RP11-545G3.1 reduces the migration and invasion of nasopharyngeal carcinoma cells. (A, B) Wound healing assay for the migration ability of CNE-2 and NP69 cells transfected with shRNA against RP11-545G3.1. (C, D) Transwell assay for the invasive ability of CNE-2 and NP69 cells after transfection with shRNA against RP11-545G3.1. ^*^*P* < 0.05; ^**^*P* < 0.01; ^****^*P* < 0.0001.

**Table 1.** The degree of nodes in the PPI network

| Name | Degree | Name | Degree |
| --- | --- | --- | --- |
| TP53 | 13 | ITGAV | 2 |
| KIF23 | 8 | HSPE1 | 2 |
| NCAPG | 7 | SIRT7 | 2 |
| RACGAP1 | 7 | CNOT7 | 2 |
| PBK | 7 | HMGA1 | 2 |
| CENPF | 6 | GINS1 | 1 |
| OIP5 | 5 | MSH2 | 1 |
| RB1 | 5 | ATP5G3 | 1 |
| CDKN1A | 5 | HSPA4L | 1 |
| KPNA2 | 5 | CARM1 | 1 |
| E2F1 | 4 | ANKRD52 | 1 |
| FGF2 | 3 | BRCC3 | 1 |
| CCND2 | 3 | HS3ST1 | 1 |
| ZNRF2 | 3 | ATG16L1 | 1 |
| KIF21A | 3 | GABARAPL2 | 1 |
| SDC4 | 3 | SCML2 | 1 |
| KIF3B | 3 | PLEKHA8 | 1 |
| TRIM37 | 2 | TRIAP1 | 1 |
| RNF144B | 2 | CPEB3 | 1 |

**S Figure 1.** The flowchart of differentially expressed mRNAs, miRNAs, lncRNAs and subsequent analysis.

**S Figure 2.** Analysis of abnormally expressed miRNAs for nasopharyngeal carcinoma in the GSE32960 dataset. (A) PCA results of 312 nasopharyngeal carcinoma (blue) and 18 normal samples (green). (B) Heat map for the correlations between samples. (C−E) Scatter plots, volcano diagram, and heat map of the differentially expressed miRNAs between nasopharyngeal carcinoma and normal specimens. Blue: down-regulation; red: up-regulation. (F) Target mRNAs of differentially expressed miRNAs by miRTarBase, TargetScan, miRDB, miRanda and miRMap databases.

**S Figure 3.** Analysis of differentially methylated genes in nasopharyngeal carcinoma in the GSE62336 dataset. (A) PCA results of nasopharyngeal carcinoma (green) and normal samples (blue). (B) Heat map for the correlations between samples. (C−E) Scatter plots, volcano diagram, and heat map of the differentially methylated sites in nasopharyngeal carcinoma. Red: hypermethylation and blue: hypomethylation. (F) Overlap of the highly expressed genes and hypomethylated genes. (G) Overlap of the lowly expressed genes and hypermethylated genes. (H) A PPI network of lowly expressed and hypermethylated genes. (I, J) GO and KEGG enrichment results of highly expressed and hypomethylated genes. (K, L) GO and KEGG enrichment results of lowly expressed and hypermethylated genes.

**S Figure 4.** Analysis of dysregulated genes shared by ceRNA regulation and DNA methylation in nasopharyngeal carcinoma. (A) Venn diagram of the up-regulated genes regulated by ceRNA and hypomethylation. (B) Venn diagram of the down-regulated genes regulated by ceRNA and hypermethylation. (C­−E) GO enrichment results of the down-regulated genes regulated by ceRNA and hypermethylation, including (C) biological processes, (D) cellular component, and (E) molecular function. (F) Pathogenic *Escherichia coli* infection pathway enriched by down-regulated genes is regulated by ceRNA and hypermethylation.

**S Figure 5.** Before and after comparison of lncRNAGSE95166 database and GSE126683 database, and quality control standards of methylation data. (A) lncRNA database GSE95166 before standardization (B) lncRNA database GSE95166 after standardization. (C) Before lncRNA GSE126683 standardization. (D) After lncRNA GSE126683 standardization. (E) Before the standardization of methylation data. (F) After the standardization of methylation data.

**S Figure 6.** Comparison of quality control standards before and after miRNA database and mRNA database. (A) Before miRNA database standardization. (B)miRNA database after standardization.(C) Before the standardization of mRNA database. (D) After the standardization of mRNA database.

**S Figure 7.** Regulatory network of miRNAs and mRNAs related to RP11-545G3.1.
